# Early postnatal microglial ablation in the *Ccdc39* mouse model reveals adverse effects on brain development and in neonatal hydrocephalus

**DOI:** 10.1186/s12987-023-00433-4

**Published:** 2023-06-09

**Authors:** Farrah N. Brown, Eri Iwasawa, Crystal Shula, Elizabeth M. Fugate, Diana M. Lindquist, Francesco T. Mangano, June Goto

**Affiliations:** 1 Division of Pediatric Neurosurgery, Cincinnati Children’s Hospital Medical Center, Cincinnati, OH USA; 2 Department of Neurosurgery, University of Cincinnati College of Medicine, Cincinnati, OH USA; 3 Department of Radiology, Imaging Research Center, Cincinnati Children’s Hospital Medical Center, Cincinnati, OH USA

**Keywords:** Microglia, Neonatal hydrocephalus, Myelination, CSF1R, Ventriculomegaly

## Abstract

**Background:**

Neonatal hydrocephalus is a congenital abnormality resulting in an inflammatory response and microglial cell activation both clinically and in animal models. Previously, we reported a mutation in a motile cilia gene, *Ccdc39* that develops neonatal progressive hydrocephalus (*prh*) with inflammatory microglia. We discovered significantly increased amoeboid-shaped activated microglia in periventricular white matter edema, reduced mature homeostatic microglia in grey matter, and reduced myelination in the *prh* model. Recently, the role of microglia in animal models of adult brain disorders was examined using cell type-specific ablation by colony-stimulating factor-1 receptor (CSF1R) inhibitor, however, little information exists regarding the role of microglia in neonatal brain disorders such as hydrocephalus. Therefore, we aim to see if ablating pro-inflammatory microglia, and thus suppressing the inflammatory response, in a neonatal hydrocephalic mouse line could have beneficial effects.

**Methods:**

In this study, Plexxikon 5622 (PLX5622), a CSF1R inhibitor, was subcutaneously administered to wild-type (WT) and *prh* mutant mice daily from postnatal day (P) 3 to P7. MRI-estimated brain volume was compared with untreated WT and *prh* mutants P7-9 and immunohistochemistry of the brain sections was performed at P8 and P18-21.

**Results:**

PLX5622 injections successfully ablated IBA1-positive microglia in both the WT and *prh* mutants at P8. Of the microglia that are resistant to PLX5622 treatment, there was a higher percentage of amoeboid-shaped microglia, identified by morphology with retracted processes. In PLX-treated *prh* mutants*,* there was increased ventriculomegaly and no change in the total brain volume was observed. Also, the PLX5622 treatment significantly reduced myelination in WT mice at P8, although this was recovered after full microglia repopulation by P20. Microglia repopulation in the mutants worsened hypomyelination at P20.

**Conclusions:**

Microglia ablation in the neonatal hydrocephalic brain does not improve white matter edema, and actually worsens ventricular enlargement and hypomyelination, suggesting critical functions of homeostatic ramified microglia to better improve brain development with neonatal hydrocephalus. Future studies with detailed examination of microglial development and status may provide a clarification of the need for microglia in neonatal brain development.

**Supplementary Information:**

The online version contains supplementary material available at 10.1186/s12987-023-00433-4.

## Introduction

Pediatric hydrocephalus is an enduring pathological condition that often requires cerebrospinal fluid (CSF) diversion surgery and significantly impacts neurocognitive and motor development. It is the most common disease process treated by pediatric neurosurgeons [1] and may be caused by intraventricular hemorrhage, spina bifida, brain infections, choroid plexus papilloma, aqueductal stenosis, or genetic mutations [2–5]. Congenital hydrocephalus, diagnosed in utero or newborns, affects 6 children per 10,000 live births [6, 7]. Congenital communicating hydrocephalus, caused by an imbalance of CSF volume control between both overproduction and malabsorption, makes up 8% of the common causes in the neonatal and infant populations according to North American centers within the Hydrocephalus Clinical Research Network (HCRN) [8, 9]. Atrophy and gliosis to periventricular white matter, including the corpus callosum, fimbria, and corticospinal tract are often described and thought to be primarily due to ventricular dilation, responsible in part, for neurocognitive and motor deficits [10]. Surgical diversion of CSF is the most common treatment for this condition; however, the cellular basis remains unsolved despite the effects leading to a lifetime of neurocognitive and neuropsychological problems in surgically treated patients. Also, about half of the children require surgical shunt revisions within 2 years [11, 12]. Hence, the immense need to develop early medical intervention persists, either in combination with surgical diversion or on its own, to potentially avert these life-long adverse symptoms.

Diffusion tensor imaging studies have shown that disturbed periventricular white matter integrity is associated with neurobehavioral deficits in pediatric hydrocephalus [13, 14] and that shunting gradually improves these deficits over time [15, 16]. The reduction in myelination and capillary densities [17, 18], as well as extracellular edema and macrophages [19, 20] are documented in earlier studies in autopsies or cerebral biopsies performed in pediatric patients with severe hydrocephalus [21]. Phagocytosed myelin was noted in symptomatic, but not in asymptomatic hydrocephalus patients [10]. Experimental animal models of neonatal hydrocephalus have described similar pathology in white matter in detail [22–32]. Also, potential causal roles of neuroinflammation have been demonstrated in the progression of hydrocephalus causing ependymal cell maturation deficit [33], glial scar formation [34, 35], or arachnoiditis [36]. Neuroinflammation is documented in the form of elevated pro-inflammatory molecules [37–40] which have the potential to serve as biomarkers to identify patients at high risk for progressive hydrocephalus.

Microglia are resident macrophages of the central nervous system (CNS) and are the primary immune cells to respond to inflammation [41]. Mature microglia and perivascular macrophages are dependent on the colony-stimulating factor 1 receptor (CSF1R) for their survival. Selective CSF1R inhibitors, including PLX3397 [42], PLX5622 [43], and BLZ945 [44], induce microglial cell death and eliminate 50–90% of microglia, within 3–7 days of treatment depending on dose and the brain area. Microglial ablation with CSF1R inhibitors in adult mice did not alter cognitive functions  [44], rather it shows therapeutic benefits in neurological disorder models by preventing microglial plaque formation in Alzheimer [43] or alleviating mechanical allodynia [46, 47]. Starting 3 days after the withdrawal of CSF1R inhibitors, new microglia begin to proliferate, migrate, and fully repopulate the mouse brain within 7 days [48]. This replacement of microglia represents a clinically feasible [54] and novel approach to temporally resolve neuroinflammation and improve cognitive decline in aging [48] and behavioral deficits/synaptic spine number in neuronal lesion injury [49] in animal models.

To investigate the roles of microglia in the pathogenesis of ventricular dilation and hypomyelination in neonatal hydrocephalus, here we evaluated the effects of PLX5622, a more potent CSF1R inhibitor than PLX3397 [50], in the *progressive hydrocephalus* (*prh*) mouse mutants [51]. The *prh* mutation identified within *coiled-coil domain-containing 39 (Ccdc39)* gene causes shorter and immotile ependymal cilia and impaired brain intraventricular CSF flow, which results in severe postnatal hydrocephalus phenotype within the first postnatal week [23]. We found pro-inflammatory (*Ccl2*^+^ *, Cd86*^+^) amoeboid-shaped microglia accumulating in the periventricular white matter [22, 24, 52]. The inhibition of NF-kB signaling using an anti-inflammatory agent, bindarit, significantly ameliorated white matter edema, hypomyelination and other neurodevelopmental deficits in the somatosensory cortex, and improved neonatal hind limb motor function of the *prh* mutant [22]. Since the microglia in immature perinatal brains have pivotal functions in brain development [48, 53], in this study we investigated whether the removal of pro-inflammatory microglia with PLX5622 could be beneficial for supporting myelination and white matter integrity, and potentially improve brain development or can induce adverse developmental defects in the *prh* mutant.

## Materials and methods

### Animal line and mouse weight

The *Ccdc39*^*prh*^ allele [51] was maintained on a mixed congenic strain background (50% CD-1 background). Heterozygous *Ccdc39*^wt/*prh*^ males and females were bred creating both homozygous *Ccdc39* mutant (*Ccdc39*^*prh/prh*^, hereafter *prh*) and wild-type (*Ccdc39*^*wt/wt*^, hereafter WT) mice. Untreated control and PLX5622 treated pups were weighed (g) on a scale every day from P3 to P20. Mice were housed in specific pathogen-free conditions, and all animal procedures were approved by the Cincinnati Children’s Hospital Medical Center Institutional Animal Care and Use Committee.

### Drug administration

PLX5622 (MedChemExpress) was dissolved in dimethyl sulfoxide (DMSO, Sigma-Aldrich) as aliquoted stock solution (50 mg/mL) and stored at -20 °C for up to four months. PLX5622 (50 mg/kg) was subcutaneously given to WT and *prh* mutant mice daily from P3 to P7. Mice were weighed before injection and the diluted PLX5622 (25 mg/mL in DMSO) was given using 0.025 mL Hamilton syringe (Hamilton company, 80222) and a 31-gauge, 12-degree angle, and 13 mm length needle (Hamilton company, 7750–22) to support injection accuracy. The injection volume ranged from 6–15 μL per animal relative to the mouse weight.

### Immunohistochemistry

P8 brains were quickly collected in phosphate-buffered saline (PBS) from *prh* and WT mice (n = 70) and immediately fixed in 4% paraformaldehyde (PFA) in PBS overnight at 4℃. P18 -P21 *prh* and WT mice (n = 24) were perfused with ice-cold PBS followed by 4% PFA, and the brain samples were fixed in 4% overnight at 4℃. All samples were washed with PBS, cryoprotected in 15% and 30% sucrose in PBS for one overnight each. From the samples immersed and frozen in NEG50 freezing medium (Thermo Fisher Scientific), 12 µm-thick sagittal cryosections were prepared and dried on slide glasses. For immunofluorescence, CTIP2-staining sections were permeabilized with 0.3% Triton X-100 (Thermo Fisher Scientific) in PBS for 30 min. After blocking in 2% normal donkey serum/0.25% Triton X-100/PBS for 1 h sections were incubated with primary antibodies of anti-rabbit IBA1 (1:500, Wako, 019–19,741), anti-goat IBA1 (1:500, Abcam, ab5076), anti-rat CTIP2 (1:1000, Abcam, ab183032), anti-rabbit CNPase (1:100, Cell Signaling, 5664), anti-rat CD86 (1:200, BD Biosciences, 553,689), anti-rabbit ApoE (1:250, Abcam, ab183597), or anti-rabbit OLIG2 (1:500, Abcam, ab136253), overnight. After washing and incubation with fluorophore-conjugated secondary antibodies (Thermo Fishers) for 2 h, the sections were washed and counterstained with DAPI (Sigma-Aldrich) for 5 min and mounted with DAPI-Fluoromount-G mounting medium (Southern Biotech).

### Microscope imaging and quantification

Images were taken with either a Nikon-Ti-E 90i upright widefield microscope with a 4×, 10×, or 20× optical lens or a confocal laser scanning microscope (Nikon A1RGaAsP inverted microscope) at 60×. Fluorescence intensity and number of cells were analyzed using the counter tool in NIS Elements software (Nikon) for the quantification of ApoE^+^IBA1^+^ and CD86^+^ IBA^+^ double -positive cells. The numbers of IBA1^+^ round amoeboid-shaped macrophages and ramified-shaped microglia were manually counted. For the quantification of CNPase signals, images were captured with a 10 × optical lens tile scan. We measured CNPase positive areas out of region of interest (ROI)s in the body of corpus callosum using the General Analysis (GA) tool in NIS Elements software. The relative CNPase^+^ area size per ROI was calculated as % and used as myelination rate. OLIG2 positive cells density was quantified using the 3 images of the corpus callosum per animal taken with 20 × optical lens, and OLIG2 and DAPI positive cells were counted using NIS automated measurement in GA tool.

### Magnetic resonance imaging and quantification

In vivo mouse brain MRI were performed on Biospec 7 T horizontal MRI system equipped with a 38 mm linear coil (Bruker Biospin, Billerica, MA). P7-9 mice were anesthetized with isoflurane and kept warm with circulating air. Temperature and respiration rate were monitored on physiological monitoring system (Small Animal Instruments, Inc. (SAI, NY)). Two respiratory-gated three-dimensional (3D) MR images were acquired using a fat-suppressed T2-weighted fast spin echo sequence (repetition time 1800 ms, echo time 80 ms, matrix 240 × 112 × 45, field of view 48 mm × 22.4 mm × 18 mm, number of echoes 20, echo spacing 10 ms, 1 average) and a fluid-sensitive sequence (fast spin echo, repetition time 2000 ms, echo time 264 ms, matrix 320 × 108 × 80, field of view 48 mm × 16.2 mm × 12 mm, number of echoes 60, echo spacing 20 ms, 4 averages) for total brain volume and ventricular volume measurement, respectively. Each series of original DICOM images were reconstructed as tiff files in Fiji software and then converted into IMARIS files via the IMARIS file converter (Bitplane Scientific Software). 3D reconstruction and volume measurements were performed using the Surfaces feature of the IMARIS software (Bitplane Scientific Software); for the total brain volume on the T2-weighted images with voxel size: x = 0.150 mm, y = 0.148 mm, z = 0.300 mm, with absolute intensity thresholding above a range of 0.58 and 2.41, and for the ventricular volumes including edematous tissue (ventricle+edema) with voxel size: x = 0.150 mm, y = 0.148 mm, z = 0.150 mm, with absolute intensity thresholding above a range of 0.13 and 0.41. The thresholding values are based on the absolute signal intensity of the gray matter. We applied the thresholding level that confirmed to clearly define the borders of the brain surface and ventricular surface in slicer view. Parenchyma volume (mm^3^) was obtained by subtracting ventricle + edema volume from total brain volume.

### Statistical analysis

All values are expressed as the mean ± standard deviation of the mean. Statistical significance of group differences between genotype (WT and *prh*) and drug treatment groups (untreated and PLX) were determined using a two-way ANOVA with Tukey’s post-hoc test for multiple comparisons. Survival data were analyzed using the log-rank procedure of Kaplan–Meier survival analysis. Body weights were analyzed by repeated measures using a two-way ANOVA, followed by Tukey’s post hoc test. All statistical computations were performed using GraphPad Prism 9, where p < 0.05 was considered statistically significant.

## Results

### PLX5622 successfully ablated neonatal microglia, including reactive amoeboid-shaped microglia in the *prh* mutant

**Fig. 1:**
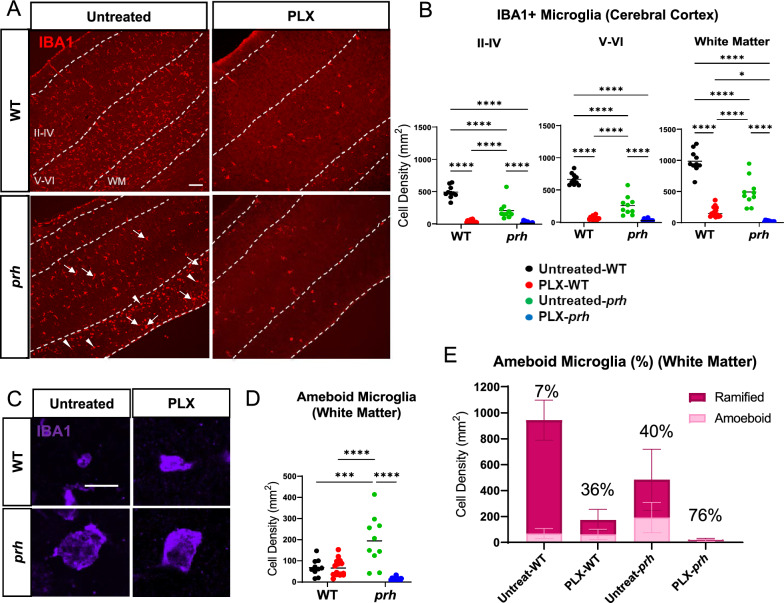
PLX5622 successfully ablated microglia in neonatal brains. **A** Low magnification 10 × images of IBA1 (red) stained in P8 WT and *prh* brains with and without PLX5622 treatment. Arrowheads: ameboid-shaped microglia, arrows: ramified microglia. Dotted lines indicate borders of cortical layers II-VI, V-VI, and white matter (WM). LV: lateral ventricle. Scale bar = 100 μm. **B** IBA1^+^ microglial densities in cortical layers II-IV (left), V-VI (middle), and white matter (right) shows that lower microglial densities in the *prh* mutants, and PLX5622 treatment significantly reduces microglial densities in all three areas. **C** High magnification (60x) images of smaller (more commonly found in WT) vs. larger (more commonly found in *prh*) amoeboid-shaped, IBA1^+^ (purple), microglia at P8. Scale bar = 10 μm. **D** Amoeboid-shaped IBA1^+^ microglia density in white matter. **E** Ratio of amoeboid-shaped microglia among total IBA1^+^ microglia in white matter. Stats: two-way ANOVA followed by Tukey’s test, n ≥ 10 in each group, ****p < 0.0001, ***p < 0.001, **p < 0.01, *p < 0.05

Considering that CSF1R inhibitors are used in clinical trials [54], we first tested the efficacy of PLX5622, a potent inhibitor of CSF1R tyrosine kinase activity (KI = 5.9 nM) [43] to ablate pro-inflammatory microglia in the early postnatal *prh* mutants. We evaluated the extent of microglial ablation in immunohistochemistry with microglial and myeloid cell marker IBA1 in the somatosensory cortex at P8 (Fig. 1A). We quantified microglial density in upper cortical layers (II-IV), lower cortical layers (V-VI), and white matter separately as the number of microglia differs in upper versus lower cortical layers in postnatal brains [55]. We found that *prh* mutants have less microglia in the grey matter relative to WTs as we reported previously (Fig. 1, Additional file 1: Table S1) [22]. After the PLX5622 treatment, the number of IBA1 ^+^ cells in the upper cortical layers (II-IV), lower cortical layers (V-VI), and white matter were all decreased in both WT and *prh* mutant. The PLX5622 treatment successfully ablated ≥ 89% microglia in all three areas above in both WT and *prh* mutants relative to untreated-WT and *prh* mutants, respectively (****p < 0.0001, two-way ANOVA followed by Tukey’s test, n = 10–15 in each group, Fig. 1B). As previously described [22, 52] pro-inflammatory microglia with a rounded amoeboid-like appearance were significantly increased in the untreated-*prh* mutant white matter (Figs. 1C, D, Additional file 1: Table S1). We found that PLX5622 treatment eliminated these amoeboid-shaped microglia (~ 95%) in *prh* mutants, represented as reduced cell density (****p < 0.0001, Fig. 1D). Among the remaining microglia, PLX5622 treatment increased the ratio of rounded amoeboid-shaped microglia to total microglia, in both WTs and *prh* mutants (Fig. 1E, Additional file 1: Fig. S1), by removing mature microglia with ramified morphology more efficiently. There was no change in amoeboid-shaped IBA^+^ cell density in WT white matter after PLX5622 (Fig. 1D), indicating most of these developing microglia in healthy P8 mice are less sensitive to PLX5622 than mature homeostatic ramified microglia. Taken together, these data indicate, early postnatal (P3-P7) systemic injection of PLX5622 effectively depletes maturing microglia in the neonatal brain, while also depleting pro-inflammatory rounded amoeboid-shaped microglia found in *prh* hydrocephalus mutants.

### Microglia profiling in neonatal hydrocephalus and of those which survived PLX5622 treatment

**Fig. 2:**
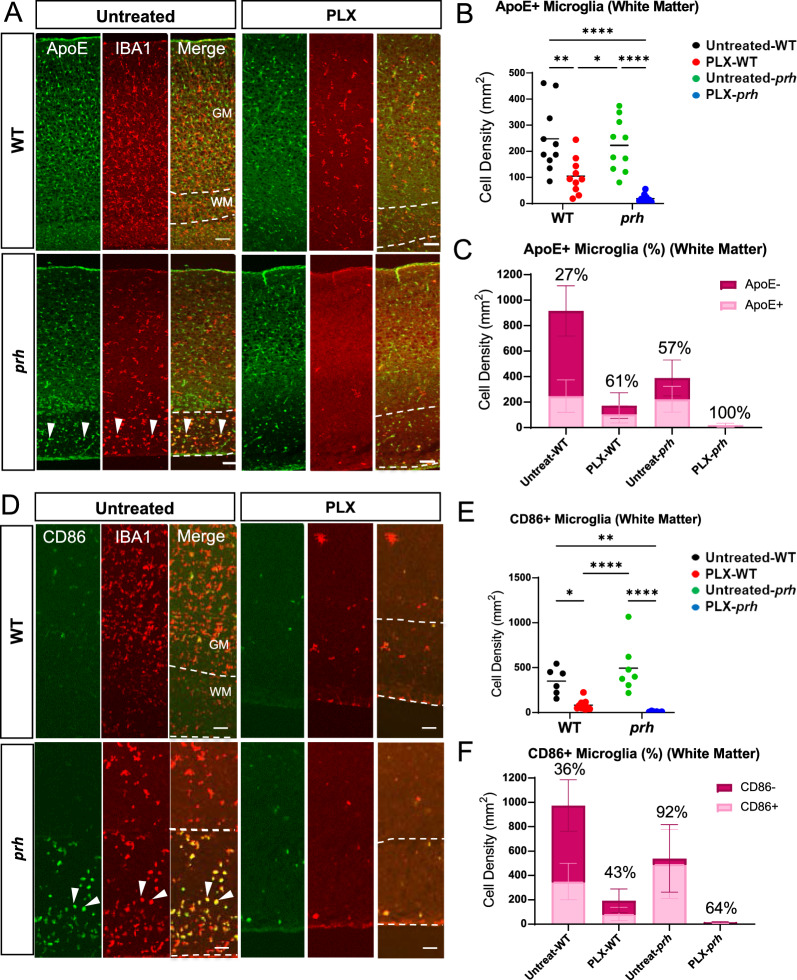
P8 microglial profiling with and without PLX5622 treatment. **A** P8 somatosensory cortex double stained with ApoE^+^ (green) and IBA1^+^ (red). Dotted lines indicate the border of grey (GM) and white matter (WM). Arrowheads: ApoE^+^ microglia. Scale bars = 100 μm. **B** The raw density of ApoE^+^ IBA1^+^ microglia is significantly reduced in PLX-treated mice compared to untreated mice. **C** Ratio of immature ApoE^+^ microglia out of total IBA1^+^ microglia in white matter. **D** P8 somatosensory cortex double stained with CD86 (green) and IBA1 (red). Dotted lines indicate grey matter layers and white matter layer. Arrowheads: CD86^+^ microglia. Scale bars = 100 μm. **E** Raw density of pro-inflammatory CD86^+^ IBA1^+^ microglia in white matter is significantly decreased in PLX-treated *prh* when compared to untreated mice. CD86^+^ IBA1^+^ microglial density is also reduced in PLX-treated WT when compared to untreated mice. **F** Ratio of pro-inflammatory CD86^+^ microglia out of total IBA1^+^ microglia in white matter. Stats: two-way ANOVA followed by Tukey’s test, n ≥ 10 in each group, ****p < 0.0001, ***p < 0.001, **p < 0.01, *p < 0.05

To further characterize microglia, we next evaluated the developmental markers of microglia in the presence of PLX5622 in WT and *prh* mutants (Fig. 2). ApoE is enriched in early postnatal microglia and injury-responsive microglia [56], disease-associated microglia [57, 58], clearance-associated microglia [59], and repopulating microglia after microglial ablation [60] and thus represent immature microglia in the healthy developing brain and disease-associated microglia in aging and brain diseases. We evaluated the ApoE^+^ and IBA1^+^ double-positive cell density as ApoE is also expressed in non-microglial cells such as astrocytes, choroid plexus epithelial cells, and endothelial cells in the developing mouse brains (http://zylkalab.org/datamousecortex). Neonatal hydrocephalus did not change the total density of ApoE^+^ microglia in white matter (Fig. 2A, B); however, it was decreased in grey matter (Additional file 1: Fig. S2A). Similarly, the ratio of ApoE^+^ microglia out of total IBA1^+^ cells was increased in white matter (Fig. 2C, Additional file 1: Fig. S2B), representing the proliferation of immature/disease associated microglia in white matter as we reported [22]. We observed, in both WT and *prh* mutants, that PLX5622 treatment decreased the ApoE^+^ microglia density, while inducing an increase in relative ratio of ApoE^+^ cells among microglia (Fig. 2C and Additional file 1: Fig. S2B) in white matter, reflecting a more prevalent ablation of mature (ApoE^−^) homeostatic and pro-inflammatory microglia. It is notable that there were no mature microglia that remained after PLX5622 treatment in the *prh* mutant’s white matter as 100% of the remaining microglia were ApoE^+^ (Fig. 2C). In grey matter, although we found less ApoE^+^ microglia density (25–250 cells per mm^2^, Additional file 1: Fig. S2A) than white matter in all groups, we found similar increase in ApoE^+^ ratio among microglia in both WT and *prh* mutant after the PLX5622 treatment (19% in untreated and 50% in PLX-WT, ***p = 0.002 in WT and 20% in untreated and 50% in PLX-*prh*, **p = 0.0015 in *prh*, respectively, Additional file 1: Fig. S2C).

In our previous reports [22, 24, 52], we observed high accumulation of activated myeloid cells expressing pro-inflammatory markers, such as MCP-1, CD68, and CD86 [61–63] in the periventricular white matter of the *prh* mutant model. Therefore, next, we evaluated the effects PLX5622 had on pro-inflammatory microglia using CD86, which is consistently regulated in pro-inflammatory conditions [64–66] (Fig. 2D-F). We quantified it only in white matter as CD86^+^ microglia were rare in grey matter in all experimental groups. As expected, the CD86^+^ microglia density was higher in mutants, and PLX5622 treatment removed ~ 98% of CD86 ^+^ microglia (Fig. 2E) and reduced percentage of CD86^+^ microglia as well (Fig. 2F, Sup Fig. 3A). Few CD86^+^ cells were also found in untreated and PLX5622-treated WT, total number of which was decreased with PLX5622 treatment (Fig. 2E). These data indicate, PLX5622 effectively depletes mature microglia and pro-inflammatory (CD86^+^) ones in the healthy and hydrocephalus neonatal brain, and mature (ApoE^−^) microglia are more sensitive to PLX5622-mediated microglial ablation.

**Fig. 3:**
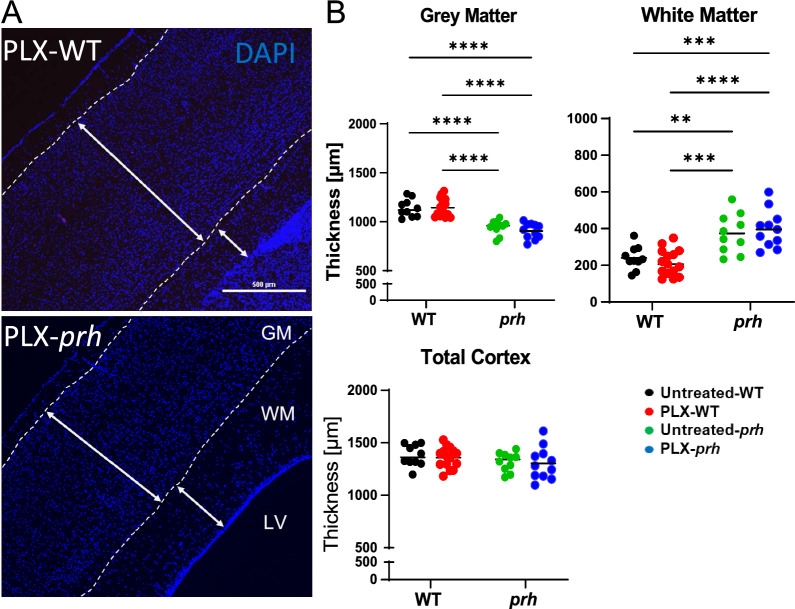
Microglial ablation does not improve grey matter thinning or white matter edema in *prh* mutants. **A** PLX-treated WT and *prh* mutant somatosensory cortex stained with DAPI (blue). Longer white double-sided arrow represents grey matter (GM) thickness, and smaller arrow represents white matter (WM) thickness. Scale bar = 500 μm. **B** Grey matter thickness. Thinner in untreated-*prh* compared, not improved by PLX5622 treatment. White matter thickness. Thicker in untreated-*prh*, which is not improved by PLX5622 treatment. PLX5622 has no effect on the thickness of white matter in WT. Total cortical thickness (grey matter thickness + white matter thickness). No statistically significant difference between treatment or genotype groups. Stats: two-way ANOVA followed by Tukey’s test, n ≥ 10 in each group, ****p < 0.0001, ***p < 0.001, **p < 0.01, *p < 0.05

### There is no improvement in white matter edema or grey matter thinning in PLX-*prh* mice

To evaluate the effects of microglial ablation on brain size and general anatomy, we measured the dorsoventral cortical thickness of cortical grey and white matter in comparable sagittal brain sections stained with DAPI (Fig. 3A). As we previously reported [22], untreated *prh* mutants have significantly thinner grey matter (****p < 0.0001 vs untreated WT, Fig. 3B, and significantly increased edematous white matter compared to untreated WT (**p = 0.0066 vs. untreated-WT Fig. 3B). Interestingly, in contrast to our study showing therapeutic benefits of the anti-inflammatory drug, bindarit [22], microglia ablation in postnatal hydrocephalus did not improve cortical thinning or white matter edema (Fig. 3B). Both control and PLX5622 treated mice within each genotype group (WT and *prh*) showed comparable thickness of grey matter, white matter, and the total cortex (= grey matter thickness + white matter thickness) in histology (Fig. 3B). These data indicate that PLX5622 mediated microglia ablation has no effect on white matter edema and thinning grey matter of *prh* hydrocephalus mutants.

### PLX5622-mediated microglial ablation negatively impacts postnatal myelination in healthy brains

**Fig. 4:**
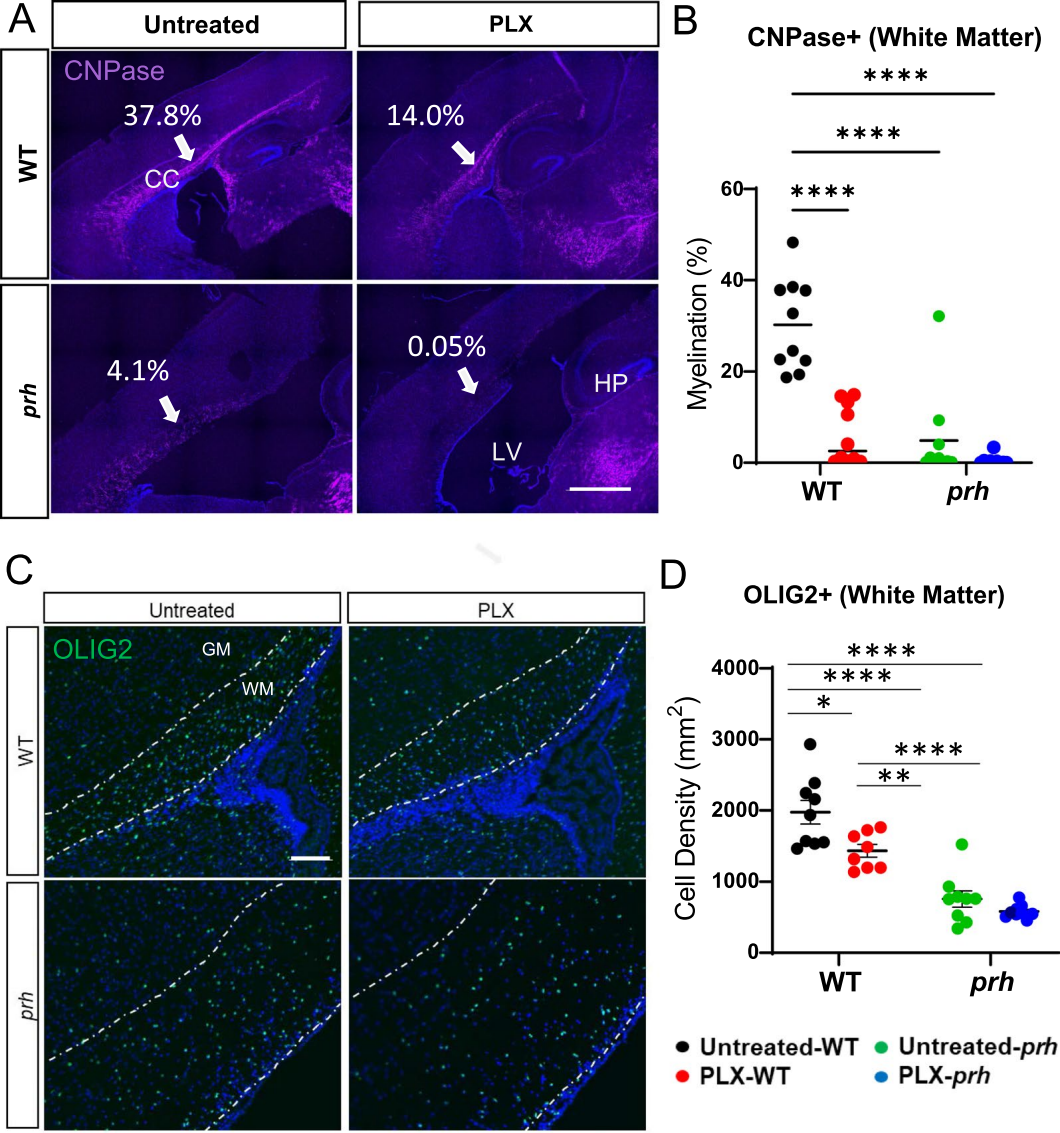
PLX5622 does not improve myelination in *prh*, rather it decreases white matter myelination in WT. **A** Low magnification 10 × images of CNPase (purple) and DAPI (blue) stained sections including somatosensory cortex in P8 WT and *prh* mice with and without PLX5622 treatment. Arrows indicate the myelination of corpus callosum where quantification is performed. Scale bar = 1000 μm. CC: Corpus callosum. LV: Lateral ventricle. HP: hippocampus. **B** Myelination density in white matter (WM), is significantly lower in PLX-treated WT, untreated-*prh*, and PLX- treated WT, compared to untreated WT at P8. **C** Pan-oligodendrocyte lineage marker OLIG2 (green) and DAPI (blue) stained in P8 WT and *prh* brains with and without PLX5622 treatment. White dotted area indicates white matter (WM). Scale bar = 100 um. **D** OLIG2 positive cells density is significantly lower in PLX-treated WT (*p = 0.011) as well as in untreated *prh* (****p = 0.0001). Stats: two-way ANOVA followed by Tukey’s test, n > 10 in B, n > 8 in D in each group, ****p < 0.0001, ***p < 0.001, **p < 0.01, *p < 0.05

It has been reported that myelination is significantly impacted early childhood hydrocephalus [67–69] and animal models of hydrocephalus. Therefore, we assessed the effects of PLX5622 microglia ablation on myelination with the mature oligodendrocyte and early myelination marker CNPase (2’, 3’ -cyclic nucleotide 3’ phosphodiesterase) (Fig. 4A, B). As we previously reported [22] CNPase staining showed a significant reduction of myelination in the P8 *prh* mutant mice (****p < 0.0001, Fig. 4A, B). We previously showed therapeutic benefits of an anti-inflammatory drug, bindarit, in hypomyelination phenotype of this mutant [22]. However, in contrast, the recovery from hypomyelination was not seen in PLX-treated *prh* animals (Fig. 4A, B). Rather, in fact, PLX5622 treatment significantly decreased myelination in WTs to the level of *prh* mutants (****p < 0.0001, Fig. 4A, B). These data indicate that early postnatal microglial ablation with PLX5622 is not beneficial for treating hypomyelination in neonatal hydrocephalus and is detrimental to early myelin maturation in healthy brains.

To further evaluate the effects of microglial ablation on oligodendrocyte maturation in early postnatal age, we quantified the pan-oligodendrocyte lineage marker OLIG2 positive cells density in these animals. Untreated *prh* had significantly less OLIG2^+^ cells density compared to untreated WT (****p < 0.0001, Fig. 4C, D) as previously reported [22], which was not rescued by PLX5622 treatment. OLIG2^+^ cells density in the corpus callosum was significantly reduced in PLX-WT relative to untreated WT (*p = 0.011, Fig. 4C, D), indicating that microglial ablation affected the overall number of oligodendrocyte lineage cells.

### PLX-treated *prh* mice have significantly larger ventricular volume

**Fig. 5:**
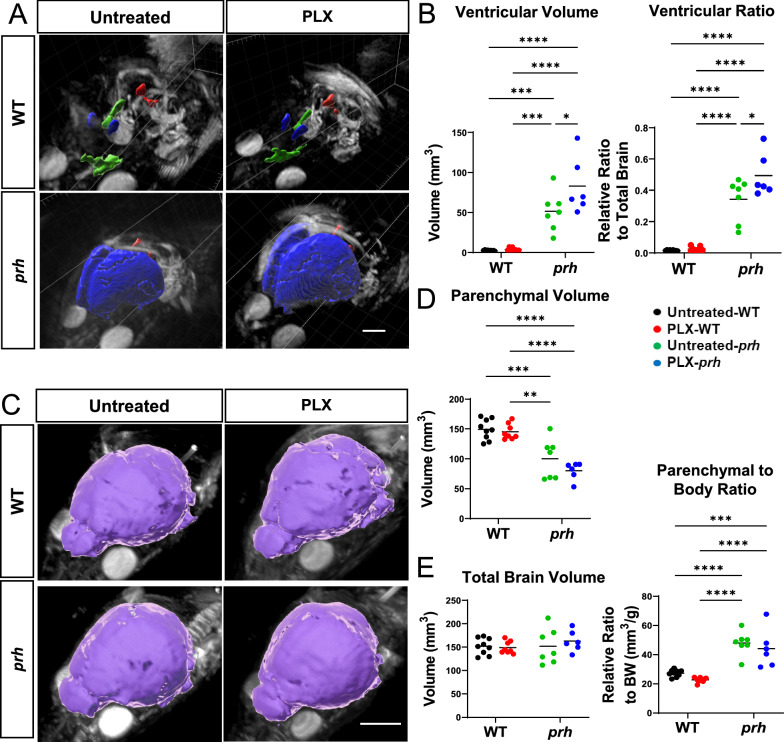
Ventricular size is enlarged, and parenchyma volume is decreased in PLX-treated *prh* mutants. **A** 3D reconstruction of fluid sensitive MR images. Blue: lateral ventricles. Green: third ventricles. Red: fourth ventricles and pineal recesses. Scale bar = 2 mm. **B** (Left) Raw ventricular volume (sum of lateral ventricles, third ventricle, fourth ventricle, and pineal recess) and its ratio to total brain volume shows enlarged ventricles in *prh* mutant animals compared to WT animals, with PLX-treated *prh* mutants also having significantly larger ventricular volume when compared to untreated-*prh*. **C** 3D reconstruction of MR images showing total brain volume. The total brain is marked purple. Scale bar = 2 mm. **D** Raw parenchyma volume, calculated by subtracting ventricular volume from total brain volume, shows *prh* mutants have significantly smaller parenchyma compared to WTs. **E** (Left) Raw total brain volume shows no significant difference among the groups. (Right) Relative brain volume to body weight (BW) shows both untreated and PLX-treated *prh* mutants have significantly higher relative brain ratio compared to WTs. Stats: two-way ANOVA followed by Tukey’s test, n > 6 in each group, ****p < 0.0001, ***p < 0.001, **p < 0.01, *p < 0.05

To see what effect, if any, PLX5622 treatment would have on the *prh* mutant’s CSF volume, we utilized three-dimensional (3D) volumetric T2-weighted MRI in ventricular volume analysis (Fig. 5A). Volumetric analysis of CSF at P7-9 as a sum of the lateral ventricles, the third ventricle, the fourth ventricle, and the pineal recess, revealed that *prh* mutants had significant ventriculomegaly compared with WT mice (***p = 0.0002 vs. untreated-WT as a raw volume (Fig. 5B left), ****p < 0.0001 vs. untreated-WT as relative ratio to the total brain volume (right), ***p = 0.0006 vs. untreated-WT as relative volume to body weight, Sup. Figure 8A, respectively). Remarkably, *prh* mutants treated from P3-P7 with PLX5622, had even further enlarged ventricular volume compared to their untreated counterparts in both raw and relative ventricular ratio to the total brain volume (*p = 0.0323 vs. untreated-*prh*, *p = 0.0246 vs. untreated-*prh*, Fig. 5B left and right respectively). We recognize that the part of edematous white matter of *prh* mutants was included in fluid-filled ventricle volume in MRI-based data, yet, it had negligible impact on our parenchymal and ventricular volume calculation.

In total brain and the subsequent parenchyma volume analyses, we found *prh* mutation or PLX treatment did not change the total brain volume (Fig. 5E). Therefore, we found that untreated*-prh *mutant had significantly smaller parenchyma volume (Fig. 5D) and ratio due to the increase in CSF. We also find that untreated-*prh* mutants have significantly smaller parenchyma volume to body weight ratio than untreated-WT mice (Fig. 5E right), which aligns with the thinner grey matter of untreated *prh* mutants in histology (Fig. 3B). In the total brain volume (= parenchyma + ventricle) evaluation, we found untreated- and PLX-treated *prh* mutants have a greater total brain volume ratio to the body weight than WT mice (****p < 0.0001 vs. untreated/PLX-treated WT, Fig. 5E right). It reflected the fact that *prh* mutants around P7-9 showed larger brain to body ratio, which is due to the enlarged cranial volume reacting to the abnormally increased CSF [22, 70]. Taken together, PLX5622-mediated microglial ablation has a negative impact on *prh* hydrocephalic brains accelerating fluid accumulation and inhibiting parenchymal growth during the early postnatal period.

### Postnatal PLX5622 treatment did not affect the growth or survival of neonatal mice

**Fig. 6:**
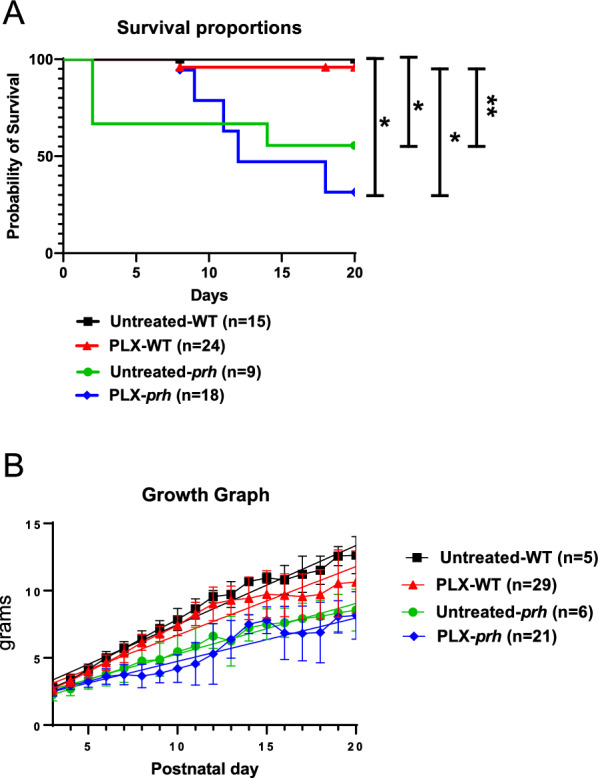
PLX-treated *prh* animals do not have survival or growth advantage over untreated-*prh* animals. **A** Survival rate of untreated-WT, PLX-treated WT, untreated-*prh*, and PLX-treated *prh* up to P20 (n ≥ 5 in each group) shows both untreated and PLX-*prh* mutants have significantly lower survival rate when compared to WTs (**p < 0.01, *p < 0.05, log-rank test). **B** Body weight analysis. There is no significant difference in the daily (P3-P20) weight in grams (g) among untreated-WT, PLX- treated WT, untreated-*prh*, and PLX- treated *prh* mice up to P20, (repeated measures of two-way ANOVA, followed by Tukey’s test)

The *prh* mutants shows lower body weight and have median survival of 10 days and typically do not survive to weaning ages [23, 51]. There were no significant differences in both survival and body weight between treatment groups within each genotype (Fig. 6). Postnatal PLX5622 treatment (P3-7) induced slightly lower body weight and 1 death out of 24 WT mice (Fig. 6B, A respectively). However, it did not affect body weight or survival of the *prh* mutants. In longitudinal growth analysis, we found significance between time and treatment/genotype groups (****p < 0.0001 time × treatment/genotype, Fig. 6B) showing that all groups grew over time regardless of the treatment, and there was no difference within the growing curve of each group, including transient change and average body weight change due to loss of sickly small mutants. These data indicate that transient microglial ablation with PLX5622 does not affect general growth and survival up to weaning age but also does not give a survival advantage in the *prh* mutant mouse line.

### Microglia fully repopulated after discontinuation of PLX5622 by P20

**Fig. 7:**
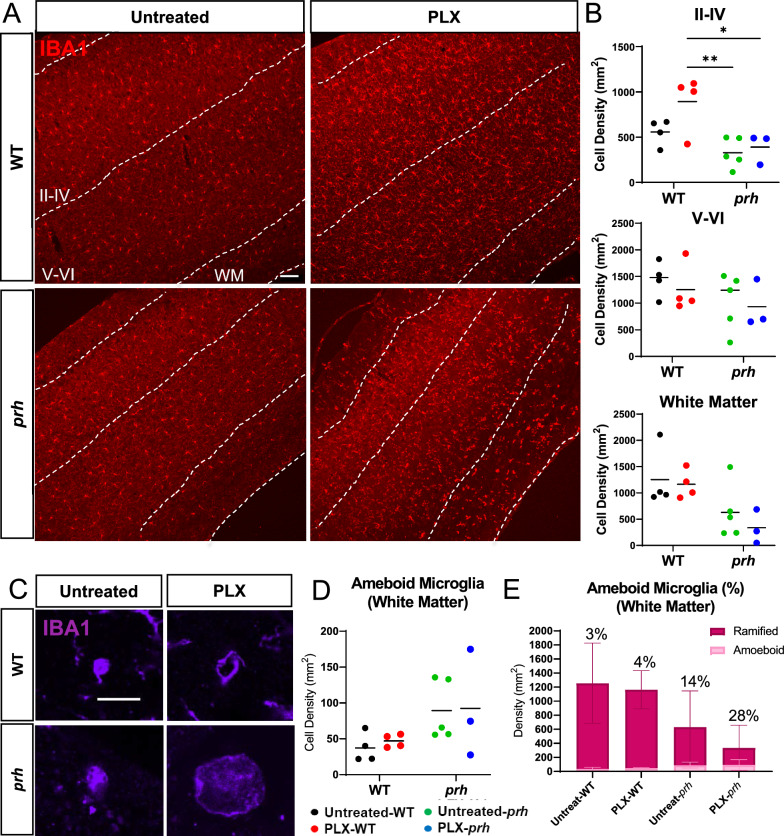
Cessation of PLX5622 treatment successfully repopulates microglia by P20 in both WTs and *prh* mutants. **A** Low magnification 10 × images of IBA1 (red) stained in P20 WT and *prh* brains with and without PLX5622 treatment. Dotted lines indicate borders of cortical layers II-VI, V-VI, and white matter (WM). Scale bar = 100 um. **B** IBA^+^ microglial densities in cortical layers II-IV (top), V-VI (middle), and white matter (bottom) shows that thirteen days post withdraw of PLX5622 treatment allows successful microglial repopulation to levels of those comparable within the same genotype group in cortical grey matter (layers II-IV and V-VI) and white matter of the somatosensory cortex (n ≥ 3 in each group). **C** High magnification (60x) images of amoeboid-shaped IBA1^+^ (purple) microglia at P20. Scale bar = 10 μm. **D** Amoeboid-shaped IBA1^+^ microglia densities in white matter are comparable between treatment and genotype groups at P20 **E** Ratio of amoeboid-shaped microglia among IBA1^+^ microglia in white matter at P20

It has been previously reported that after CSF1R inhibitor removal, microglia proliferate and differentiate from the few remaining (immature) microglia within 3 days [52, 70, 73] and fully repopulate the adult and neonatal brain within 7–14 days [45, 53, 71]. Therefore, we analyzed microglia and anatomical phenotypes > 11 days after the last PLX5622 dosing and analyzed the effect of microglial repopulation at P18-20 (Fig. 7A). In the *prh* mutants that survived to P18-21, macro phenotypes include large doming of the head along with severe ventricular enlargement (n > 5). These *prh* mutant mice are also visibly smaller (sometimes seeming malnourished) than their WT littermates, but microglial density was not much different from WTs (Fig. 7). Comparing to ablation at P8, we found that microglia density was nearly fully recovered (~ 98%) in all layers (II-IV, V-VI, and white matter) of the somatosensory cortex in juvenile WTs (Fig. 7B top, middle, & bottom). In the *prh* mutants, microglial density was also recovered in all layers *comparable* to the untreated mutant levels (n = 3,4). (Fig. 7B). We also increased percentage of rounded amoeboid-shaped microglia among total IBA1^+^ microglia stayed moderately higher in the mutant groups than the WTs, it was significantly higher in *prh* mutants with repopulated microglia compared to WT groups (Additional file 1: Fig. S4A). Altogether, aligned with previous reports, we found microglia repopulate the postnatal brain within 2 weeks after the drug removal. The repopulation of microglia in *prh* mutants did not change the density and shapes of microglia in juvenile mutant brain.

### Repopulated microglia profiles 13 days after PLX5622 removal

**Fig. 8:**
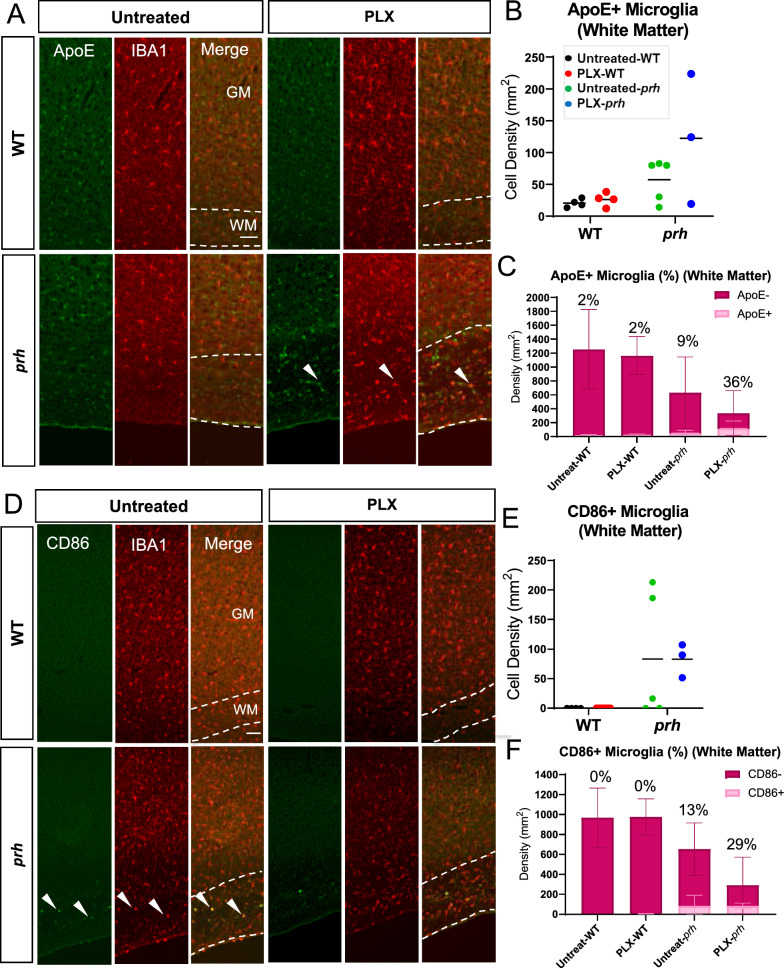
Characteristics of microglia after repopulation in the white matter of WT and *prh* brains at P20*.* **A** P20 somatosensory cortex double stained with ApoE (green) and IBA1 (red). Dotted lines indicate the border of grey (GM) and white matter (WM). Arrowheads: ApoE^+^ microglia. **B** The raw density of immature ApoE^+^ Iba1^+^ microglia in white matter shows no significant difference in density between treatment or genotype groups in white matter at P20 (two-way ANOVA followed by Tukey’s test, n ≥ 3 in each group). **C** Ratio of immature ApoE^+^ microglia out of total IBA1^+^ microglia in white matter. **D** P20 somatosensory cortex double stained with CD86 (green) and IBA1 (red). Dotted lines indicate grey matter (GM) layers and white matter (WM) layer. Arrowheads: CD86^+^ microglia. **E** Raw density of pro-inflammatory CD86^+^ IBA1^+^ microglia in white matter shows no significant difference between treatment and genotype groups at P20 (two-way ANOVA followed by Tukey’s test, n ≥ 3 in each group). **F** Ratio of pro-inflammatory CD86^+^ microglia out of total IBA1^+^ microglia in white matter. Scale bars: 100 μm.

We next evaluated the status of microglia after withdrawal of PLX5622 in WTs and *prh* mutants at P18-21 (Fig. 8) with ApoE and CD86. Through the staining of immature microglial marker ApoE (Fig. 8A-C), we observed no statistically significant difference in the density of ApoE^+^ microglia in neither the white matter (10–230 cells per mm^2^, Fig. 8B) or grey matter (5–39 cells per mm^2^, Additional file 1: Fig. S5A) in all groups of the juvenile brains; although, both some treated and untreated-*prh* mutants showed higher ApoE^+^ microglia density and ratio of white matter microglia relative to WTs (Fig. 8B, C respectively, Two-way ANOVA, main factor “genotype” F (1, 12) = 8.014, *p = 0.0152, Additional file 1: Table S1). In fact, the percentage of immature ApoE^+^ microglia in PLX-treated *prh* mutants in the white matter remained high compared to untreated/PLX-treated WT mice and untreated-*prh* mutants (**p = 0.0040 vs. untreated-WT; **p = 0.0047 vs. PLX-WT; *p = 0.0460 vs. untreated-*prh*, Fig. 8C, Additional file 1: Figure S5B).

Pro-inflammatory CD86^+^ microglia were not found in the juvenile WT groups with or without microglial repopulation in the white matter (Fig. 8D), whereas it was found in *prh* mutants with and without microglial repopulation (0–225 cells per mm^2^, Fig. 8E). Therefore, the hydrocephalus phenotype affected the presence of CD86^+^ microglia in white matter (Two-way ANOVA, main factor “genotype” F (1, 15) = 9.434, **p = 0.0078). Although, the total CD86^+^ cell density was not different between untreated-*prh* vs PLX-*prh*, percentages of CD86^+^ microglia in white matter were higher in PLX-*prh*, but not in untreated-*prh*, compared to WT mice (**p = 0.0076 vs. untreated-WT; **p = 0.0035 vs. PLX-WT, Fig. 8F, Additional file 1: Figure S6A). This data indicated that repopulated microglia does not only induce the relatively immature status of microglia (Fig. 2C) but may exacerbate the immature and pro-inflammatory status by repopulation in developing hydrocephalic brains.

### Myelination recovery 13 days after PLX5622 cessation

**Fig. 9:**
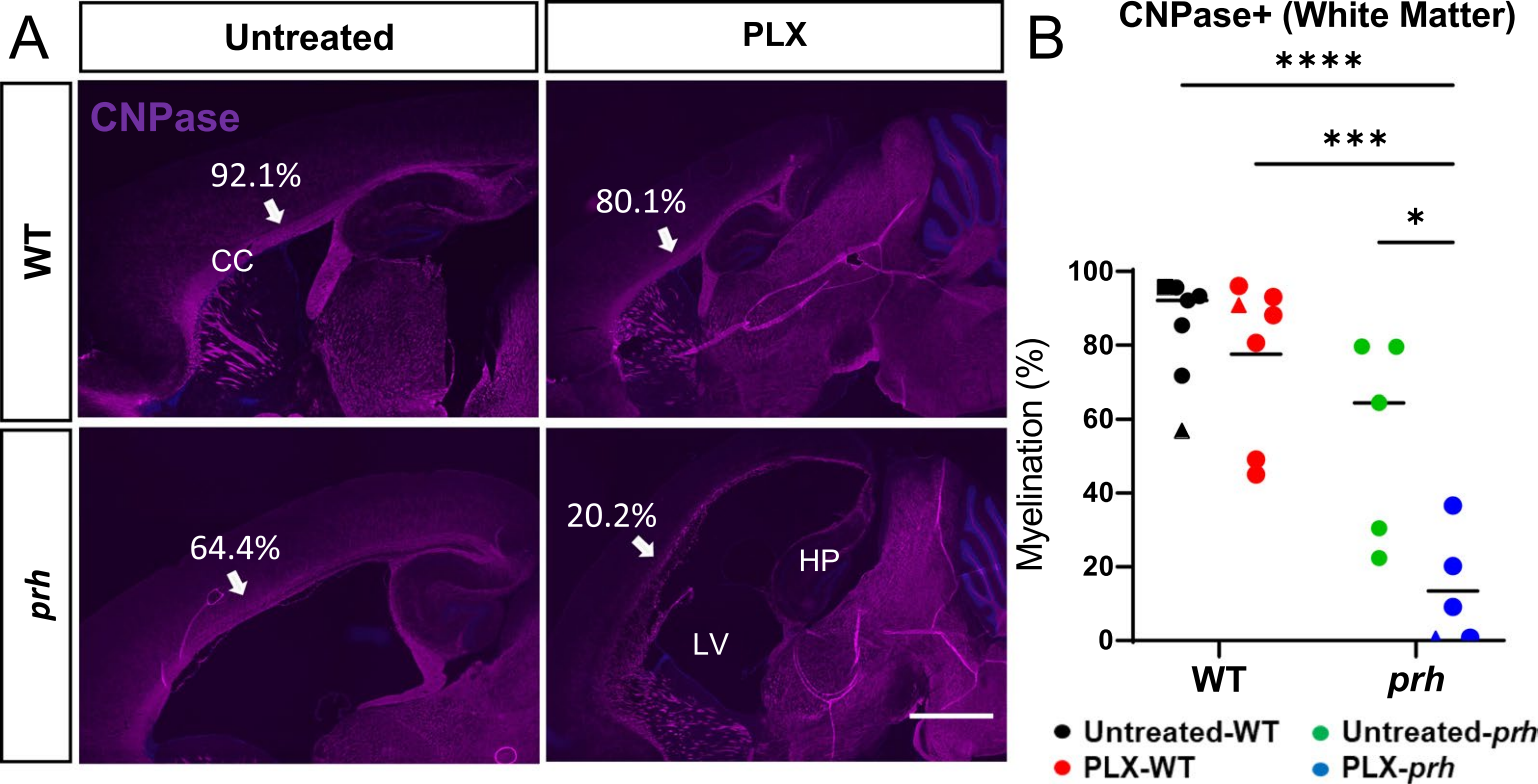
Myelination is recovered in WTs after microglia repopulation at P20, but it worsens hypomyelination phenotype in *prh* mutants. **A** Low magnification 4 × images of CNPase (purple) and DAPI (blue) stained in P20 WT and *prh* brains with and without PLX5622 treatment. Arrows indicate the myelination in corpus callosum (CC) where myelination is quantified. Scale bar = 1000 μm. LV: Lateral ventricle. HIP: Hippocampus, **B** Quantification of myelination density at P18-21 shows that myelination is comparable between PLX-treated WT mice within thirteen days after withdrawal of PLX5622 treatment. PLX-treated *prh* animals has significantly lower myelination density than untreated *prh* mutants. P18 (triangles), P20 (circles), and P21 (rectangle). Stats: two-way ANOVA followed by Tukey’s test, n > 5 in each group, ****p < 0.0001, ***p < 0.001, **p < 0.01, *p < 0.05

Statistically, the current PLX treatment and withdrawal mildly affects the myelination level of the corpus callosum at P18-21 (Two-way ANOVA, F (1, 20) = 8.680 **p = 0.0080), After withdrawing PLX5622 for 11–13 days, myelination was recovered in juvenile WT mice to comparable levels as untreated WT mice (Fig. 9, *p* = 0.9275). The untreated *prh* mutant showed numerically, but not statistically, lower myelination than untreated WT in post-hoc analysis with Tukey’s test (Fig. 9: Untreated-WT 84.4 ± 13.7%, n = 7; untreated-*prh*, 55.0 ± 24.3%, n = 5, Two-way ANOVA, post hoc *p* = 0.0919). The p-value of 0.0919 in post hoc test was considered a statistical trend that in two-group comparison between them with student t-test have reached significance (t-test; *p = 0.037). PLX-mediated ablation and repopulation of microglia significantly affected the myelination of *prh* mutants (Fig. 9: PLX-*prh*, n = 6, 13.5 ± 13.6%, Two-way ANOVA, post hoc *p = 0.0167). In general, these data indicated that the PLX5622 treatment (P3-7, 50 mg/kg) in the *prh* mutant did not improve myelination, if not worsened. Indeed, microglia repopulated *prh* had statistically significantly lower levels of myelination than WT mice (****p  < 0.0001 vs. untreated-WT; ***p < 0.001 vs. PLX- WT, Fig. 9B), which implied that early PLX5622 treatment left a notable negative impact on myelination in the neonatal hydrocephalic brain. This data is indicative of the slower progress of myelination in *prh,* which is not improved by the microglial repopulation. Thus, myelination in the healthy neonatal brain can be recovered after microglial ablation. However, in case of prolonged hydrocephalus and a low-grade pro-inflammatory state, this recovery does not occur.

## Discussion

Neuroinflammation is commonly described in perinatal hydrocephalus patients and animal models as a form of elevated pro-inflammatory cytokines in the CSF or gliosis, despite little involvement of peripheral immune cell infiltration [10, 19–32]. In this study, we attempted to deplete “pro-inflammatory” microglia in the *prh* mutant and tested its potential benefit for supporting prenatal myelination and lessening the severity neonatal hydrocephalus. We used a potent and selective CSF1R inhibitor, PLX5622, to achieve faster and more efficient microglial ablation within 3 days of treatment than PLX3397 [73]. We found substantial (89%) microglial ablation in both pro-inflammatory and homeostatic microglial in a robust neonatal hydrocephalus mouse model as well as in WT. However, the elimination of microglia in the neonatal hydrocephalus brain caused no improvement of edema or grey matter thinning, rather it worsened CSF volume and cortical thinning in P7-9*,* and left hypomyelination phenotype in juvenile (P18-21) mutant. We report that PLX5622 is a reliable method for ablating microglia in the early postnatal mouse brain; however, it can be harmful during the neonatal stage in both WT and in hydrocephalic mouse brains. Although our data is limited by small sample size in juvenile mutants, PLX5622-mediated microglial ablation did not bring positive outcomes in neonatal hydrocephalus. The delayed myelination in the PLX-treated WT brain highlight the critical functions of myeloid cells in early postnatal myelination, as it was previously shown in the prenatal [53], early postnatal CSF1R inhibitors [44] application, or genetic loss of CSF1R [44, 74, 75], where parenchymal microglia but not brain boarder macrophage ablated.

Neonatal hydrocephalus has significant negative effects on perinatal myelination in patients [76] and animal models [77] regardless of its etiology. Hydrocephalus-induced hypoxia reduces cerebral blood flow, causing neurodevelopmental delay or nerve/brain injury, which may directly or in-directly affect oligodendrocytes and their precursors [30, 78]. The pro-inflammatory response of the microglia may also indirectly affect oligodendrocyte development or function and mediate the hypomyelination phenotype [79] in neonatal hydrocephalus. Consistent with our current and previous findings in this model, elevated pro-inflammatory cytokine levels and glial activity are documented in the CSF and brains of patients with neonatal hydrocephalus [38–40, 80–82]. Although we did not study the direct effect of PLX5622 on hypoxia/ischemia, future studies of analyzing microglia-dependent pro-inflammatory cytokines in hydrocephalus may elucidate further mechanisms disturbing myelinogenesis in neonatal hydrocephalus. Our findings did not support the noxious effects of pro-inflammatory microglia in neonatal hydrocephalus, rather highlighted the importance of homeostatic brain myeloid cells in myelination that are also affected by PLX5622. The process of myelin biogenesis, including oligodendrocyte maturation and myelination, occurs in the third trimester of gestation in humans [67–69] and the first 1–2 weeks of the postnatal period in rodents [83] along with microglial maturation in grey matter and the presence of amoeboid-shaped axon tract-associated microglia (ATM) in white matter [84–86]. Therefore, our model may reflect how perinatal myelinogenesis gets perturbed by loss of microglial functions in hydrocephalus. Myeloid cells in healthy developing white matter are essential for driving myelination via region-dependent roles in oligodendrocyte survival, differentiation, and myelin production [79]. The ATM provide trophic factors, such as IGF1 that can promote the maturation and survival of oligodendrocytes [87]. Recent studies have highlighted specific functions of perinatal microglia in eliminating nascent myelin deposits [88] and excess oligodendrocyte precursor cells (OPCs) [89], that are critical for myelination. In fact, microglia ablation with prenatal PLX5622 [53], or postnatal BLZ945, or genetic loss of *Csf1r* gene [44] results in impaired myelination in early postnatal mice. Therefore, further studies investigating the developmental status of oligodendrogenesis in the *prh* mutant may elucidate the impact of neonatal hydrocephalus on myeloid cell guided myelination.

We previously reported that the anti-inflammatory NF-kB inhibitor, bindarit, had therapeutic benefits in promoting myelination in the *prh* model [22]. Bindarit reduced pro-inflammatory amoeboid-shaped microglia and rescued the cell density of homeostatic microglia (22). In contrast, the neonatal PLX5622 treatment removed both homeostatic and pro-inflammatory myeloid cells and left mostly immature ApoE^+^ ones with no ramified processes throughout the cortical and subcortical regions in the *prh* mutants and WTs evaluated at P8. Therefore, our data suggests that loss of resident myeloid cells and their homeostatic functions in the neonatal period, rather than a gain of pro-inflammatory microglia, may greatly contribute to hypomyelination in neonatal hydrocephalus.

Motor and neuropsychological phenotypes, such as hyperactivity, uncoordinated movements, epilepsy, spasticity, or anxiolytic-like behaviors are seen in rodents [53, 74, 90] and humans [75] without microglia during brain development. These may reflect the lack of microglia-dependent oligodendrocyte functions [79] and/or neural circuit formation early in life [91]. As neonatal hydrocephalus causes similar motor and neurological problems, the development of therapeutic methods to enhance the survival or development of perinatal homeostatic microglia promises to be an attractive approach to better support brain development in neonatal hydrocephalus.

In alignment with a previous report using BLZ945 [44], here we found that microglial ablation in healthy newborn mice affected myelination in the corpus callosum of all PLX5622 treated mice that were evaluated at P8. The effects of microglia on oligodendrocyte maturation or myelination are likely specific to development as microglial ablation in the adult brain does not affect oligodendrocyte and its precursor cell densities [44] nor cognitive function [43, 45, 92]. Different CSF1R inhibitors may directly affect oligodendrocyte and/or OPC survival; however, it is likely that the PLX5622 dose we used here (50 mg/kg) has a minimal direct effect on the oligodendrocytes due to its higher specificity to CSF1R compared to other kinases such as PDGFRs / KIT (IC_50_ CSF1R: 10 nM: IC_50_ PDGFRβ, PDGFRa, KIT: > 1 μM) [93] or PLX3397 (IC_50_ CSF1R: 20 nM; IC_50_ KIT: 10 nM), and similar levels to BLX945 (IC_50_ CSF1R: < 1 nM; IC50 PDGFRβ: > 1 μM) [94]. Moreover, previous studies show that cultured oligodendrocytes and OPCs are tolerant to CSF1R inhibitors [73]. Therefore, the direct effects of PLX5622 on oligodendrogenesis via modulating their PDGFRa or other kinases in neonatal mouse brains are likely minimal. Of note, our initial attempt to use PLX3397 (40 mg/kg, subcutaneously administered three times at P2, P4 & P6, analysis at P8, data not shown) to deplete microglia in neonatal mice was unsuccessful. It resulted in subtle and variable microglial ablation, which likely reflects its lower potency compared to that of PLX5622 reported in adult microglial ablation [95].

Recent studies indicate that microglia can repopulate through the few immature Nestin +microglia that survive CSF1R inhibitor treatment in adults [71, 72] and neonatal [53] brains. The microglia repopulation speed is proportional to the extent of microglial depletion [96]. Although we did not evaluate the Nestin levels in our remaining or repopulated microglia, we found full repopulation of microglia 11–13 days after the withdrawal of PLX5622 in juvenile WT and *prh* mice and speculate that cortical repopulating microglia are proportional to the number of surviving immature microglia and respond to hydrocephalus. In studies of adult brain disease models, such as Alzheimer’s disease [45] or neuronal injury [49], the microglial replacement had beneficial effects on cognitive functions [48] or behavioral deficits [49]. However, in our current study, although it was not statistically significant, the higher presence of cellular and molecular features of immature/activated microglia, i.e., amoeboid/round shape, expressing ApoE and CD86, and lower presence of mature ramified microglia. Therefore, we conclude that microglial replacement in the neonatal hydrocephalus may not improve myelination. It rather appears to have long detrimental effects, further impairing myelination during the first 3 postnatal weeks. Remarkably, we found that myelination was able to fully recover to the normal level in the juvenile WT mice after transient neonatal PLX5622 treatment, which suggested temporal chemical microglial ablation in healthy developing brain may have minimal long-term effects in brain development. At P20, 13 days after drug withdraw, microglia densities are comparable to untreated WT and *prh* mutant brains, respectively, in large. However, due to the high mortality of the *prh* mutants, the small number of P20 animals used in this study remains the limitation of this study, and the trend of increased ameboid microglia in the both untreated and PLX-treated mutant could be further addressed. Taken together, our findings suggest critical developmental and functional roles of microglia, particularly in myelination, in neonatal hydrocephalus.

Previous studies have reported causal effects of neuroinflammation in progressing ventricular enlargement via activating SPAK1-NKCC1 signal-mediated hypersecretion of CSF from the choroid plexus in the posthemorrhagic hydrocephalus model [99] or via impairing ependymal maturation and ciliogenesis in GFAP.tTA/(tetO)7.IKK2-CA [33] and Vps35-knockout mouse models [42]. Conversely, in our *prh* hydrocephalus mouse model, removal of microglia-mediated inflammation and perinatal microglia by PLX5622 did not improve, but rather, accelerated the hydrocephalus phenotype. In our study, we found that the periventricular edema was worsened by PLX5622 treatment. As shown in two recent studies, subtypes of microglia also have roles in brain vascular function; inflammation can recruit vessel-associated microglia that disrupts blood–brain barrier [100], and capillary-associated microglia are important for the reactivity of capillary control of cerebral blood flow [101]. Therefore, further investigation of the cause of periventricular edema and its contribution to the ventricular dilation in PLX5622 treated hydrocephalus model is important.

Recently, it was reported that hydrocephalus caused by the loss of the ependymal Vps35 gene is reversible with PLX3397-mediated microglial ablation [42]. However, in our study, we were unsuccessful in ablating a large number of microglia in neonatal brains with PLX3397. Further studies on the differences in the chemical properties of PLX3397 or sensitivity to CSF1R inhibitors in different strains of mice may also be needed.

## Conclusion

In the current study, we demonstrate that postnatal ablation of microglia causes ventricular enlargement and does not improve hypomyelination in hydrocephalus mutant mice. Neonatal ablation also did not contribute to improving myelination at the juvenile stage. Therefore, our findings demonstrate the importance of supplying healthy microglia for the treatment of neurodevelopmental problems in neonatal hydrocephalus. Our current study shows microglia are necessary for managing CSF and parenchymal volume and for supporting myelination in neonatal hydrocephalus. As we continue to pursue better neurological outcomes for hydrocephalus patients it will be important to evaluate and understand the molecular effects of drugs like PLX5622 in healthy brain development. Appropriate translational therapeutic targets that do not completely ablate the microglial response should be considered in future work.

## Supplementary Information

**Additional file 1: Figure S1.** PLX treatment increases the percentage of amoeboid-shaped microglia in white matter at P8. **A** PLX-treated mice have a higher percentage of rounded amoeboid-shaped microglia compared to untreated mice at P8, quantified using IBA1^+^ microglial images taken with 10 × objective lens. **Figure S2.** PLX increases percentages but reduces density of immature ApoE^+^ microglia in grey matter at P8. **A** ApoE^+^ microglial density in cortical grey matter density at P8, quantified using ApoE and IBA1 double-stained sections taken with 10 × objective lens. Untreated *prh*, PLX-WT and PLX-*prh* have significantly lower raw densities of immature ApoE^+^ IBA1^+^ microglia than untreated-WT at P8. **B** Both PLX-WT and PLX-*prh* have a significantly higher percentage of ApoE^+^ IBA1^+^ immature microglia in white matter after PLX5622 treatment compared to untreated-WT and untreated-*prh,* respectively, and 100% of microglia that survives PLX5622 treatment is ApoE^+^. 33 **C** Both PLX-WT and PLX-*prh* have a significantly higher percentage of ApoE^+^ IBA1^+^ immature microglia in grey matter after PLX5622 treatment compared to untreated-WT and untreated-*prh,* respectively*.*. **Figure S3.** Untreated-*prh* have significantly higher percentages of pro-inflammatory CD86^+^ microglia than WTs and PLX-*prh* at P8. **A** Percentage of CD86^+^ IBA^+^ double-positive microglia in white matter at P8, quantified using CD86 and IBA1 double-stained sections taken with 10 × objective lens. The percentage of CD86^+^IBA1^+^pro-inflammatory microglia in untreated *prh* is significantly increased in the white matter compared to untreated-and PLX-WT. PLX5622 treatment significantly reduces the percentage of CD86^+^IBA1^+^microglia in *prh*. **Figure S4.** Microglial repopulation increases percentage of amoeboid-shaped microglia in white matter of *prh* mutants at P20. **A** PLX-*prh* mutants have a significantly higher percentage of amoeboid-shaped microglia compared to untreated and PLX-WT at P20 after microglial repopulation, quantified using IBA1stained animals, with 10 × objective lens. **Figure S5.** Microglial repopulation increases percentages of immature ApoE microglia in white matter of *prh* at P20. **A** There is no statistically significant difference among groups in the percentages of immature ApoE^+^ IBA1^+^ double-positive microglia out of total IBA1^+^ microglia in grey matter at P20 after repopulation, measured from low power 10 × cortex photos of ApoE and IBA1 double-stained animals. **B** PLX-*prh* mutants have a significantly higher percentage of immature ApoE^+^ IBA1^+^ microglia in white matter than untreated-WT, PLX-WT, and untreated *prh* after repopulation at P20. **C** Percentage of immature ApoE^+^ IBA^+^ double-positive microglia in grey matter at P8, measured from low power 10 × cortex photos of ApoE and IBA1 double-stained animals. There is no statistically significant difference among groups in the percentage of immature ApoE^+^ IBA^+^ double-positive microglia in grey matter at P20 after repopulation. **Figure S6.** PLX-*prh* have significantly higher percentages of pro-inflammatory CD86^+^ microglia after repopulation at P20. **A** Percentage of CD86^+^IBA^+^ double-positive microglia out of total IBA1^+^ microglia in white matter at P20 after repopulation, is significantly increased in PLX-*prh,* compared to untreated-WT and PLX-WT. **Figure S7.** *Prh* mutants weigh less than WTs at P8. **A** Body weight in gramsof the mice at P8. Both untreated-prh and PLX-treated prh weigh significantly less than WTs. **Figure S8.** Ventricle and parenchyma volume at P8 normalized to body weight. **A** Ventricular volumes normalized to body weight in grams at P8 shows that untreated-*prh* and PLX-*prh* mutants have significantly enlarged ventricles compared to WT mice. **B** Parenchyma volume normalized to body weight at P8 shows that PLX-treated mice have significantly smaller parenchyma compared to their untreated control groups. **Table S1.** The two-way ANOVA sstatistical analysis results.

## Data Availability

The datasets used and/or analyzed during the current study are available from the corresponding author on reasonable request.

## Abbreviations

*Prh*: Progressive Hydrocephalus
PLX5622: Plexxikon 5622
WT: Wild Type
P: Postnatal Day
DMSO: Dimethyl Sulfoxide
WM: White Matter
GM: Grey Matter
