# Supplementary material for: Early postnatal microglial ablation in the Ccdc39 mouse model reveals adverse effects on brain development and in neonatal hydrocephalus
Source: Fluids Barriers CNS. 2023 Jun 9;20:42. doi: 10.1186/s12987-023-00433-4 (PMC10251678; doi:10.1186/s12987-023-00433-4)
Supplement: Supplementary file 1 — Additional file 1: Figure S1. PLX treatment increases the percentage of amoeboid-shaped microglia in white matter at P8. A PLX-treated mice have a higher percentage of rounded amoeboid-shaped microglia compared to untreated mice at P8, quantified using IBA1+ microglial images taken with 10 × objective lens. Figure S2. PLX increases percentages but reduces density of immature ApoE+ microglia in grey matter at P8. A ApoE+ microglial density in cortical grey matter density at P8, quantified using ApoE and IBA1 double-stained sections taken with 10 × objective lens. Untreated prh, PLX-WT and PLX-prh have significantly lower raw densities of immature ApoE+ IBA1+ microglia than untreated-WT at P8. B Both PLX-WT and PLX-prh have a significantly higher percentage of ApoE+ IBA1+ immature microglia in white matter after PLX5622 treatment compared to untreated-WT and untreated-prh, respectively, and 100% of microglia that survives PLX5622 treatment is ApoE+. 33 C Both PLX-WT and PLX-prh have a significantly higher percentage of ApoE+ IBA1+ immature microglia in grey matter after PLX5622 treatment compared to untreated-WT and untreated-prh, respectively.. Figure S3. Untreated-prh have significantly higher percentages of pro-inflammatory CD86+ microglia than WTs and PLX-prh at P8. A Percentage of CD86+ IBA+ double-positive microglia in white matter at P8, quantified using CD86 and IBA1 double-stained sections taken with 10 × objective lens. The percentage of CD86+IBA1+pro-inflammatory microglia in untreated prh is significantly increased in the white matter compared to untreated-and PLX-WT. PLX5622 treatment significantly reduces the percentage of CD86+IBA1+microglia in prh. Figure S4. Microglial repopulation increases percentage of amoeboid-shaped microglia in white matter of prh mutants at P20. A PLX-prh mutants have a significantly higher percentage of amoeboid-shaped microglia compared to untreated and PLX-WT at P20 after microglial repopulation, quantified using IBA1stained [file 12987_2023_433_MOESM1_ESM.pptx]

## Slide 1
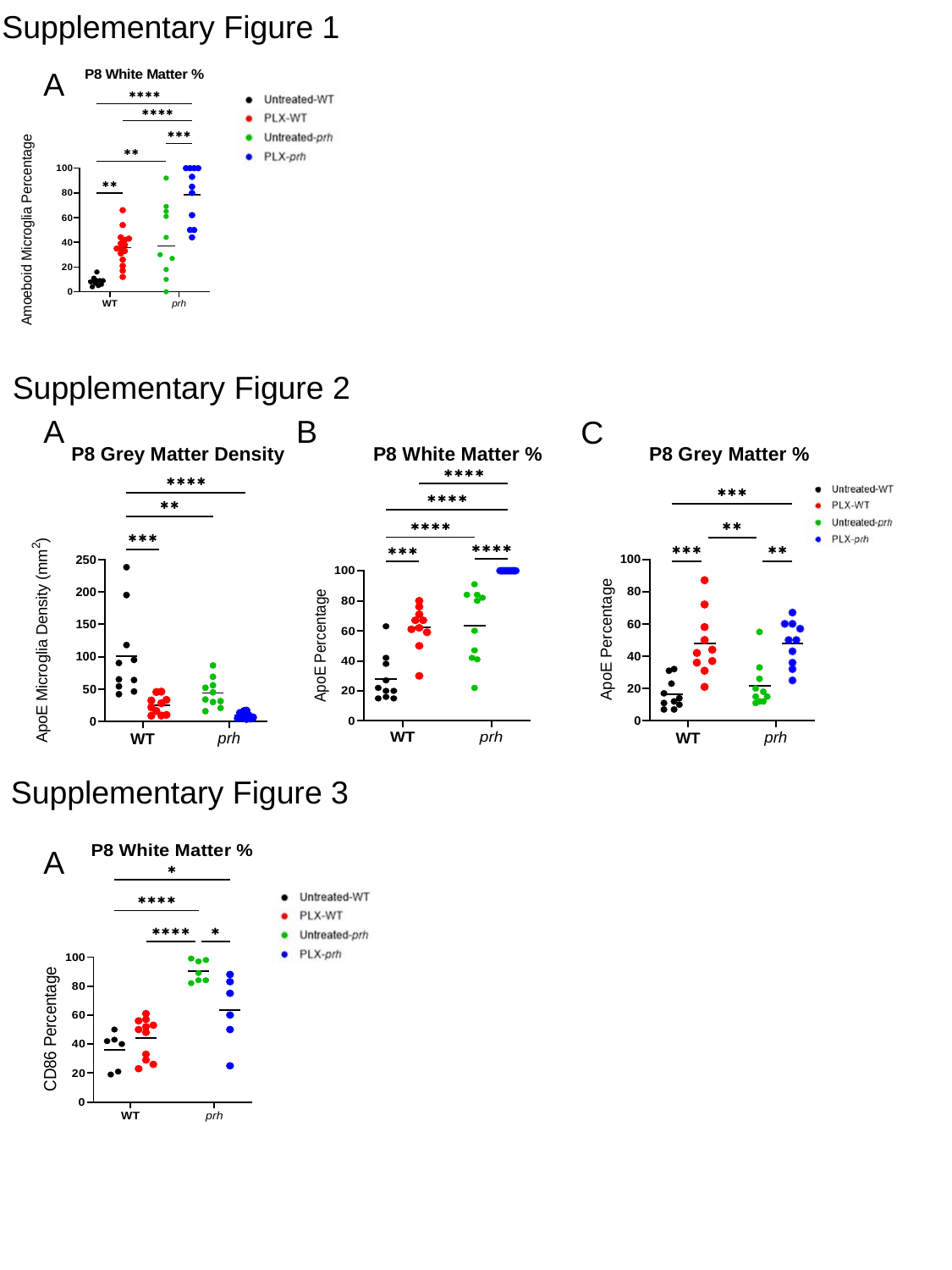

Supplementary Figure 1
A
Supplementary Figure 2
A
B
C
P8 Grey Matter Density
P8 White Matter %
P8 Grey Matter %
Supplementary Figure 3
A

## Slide 2
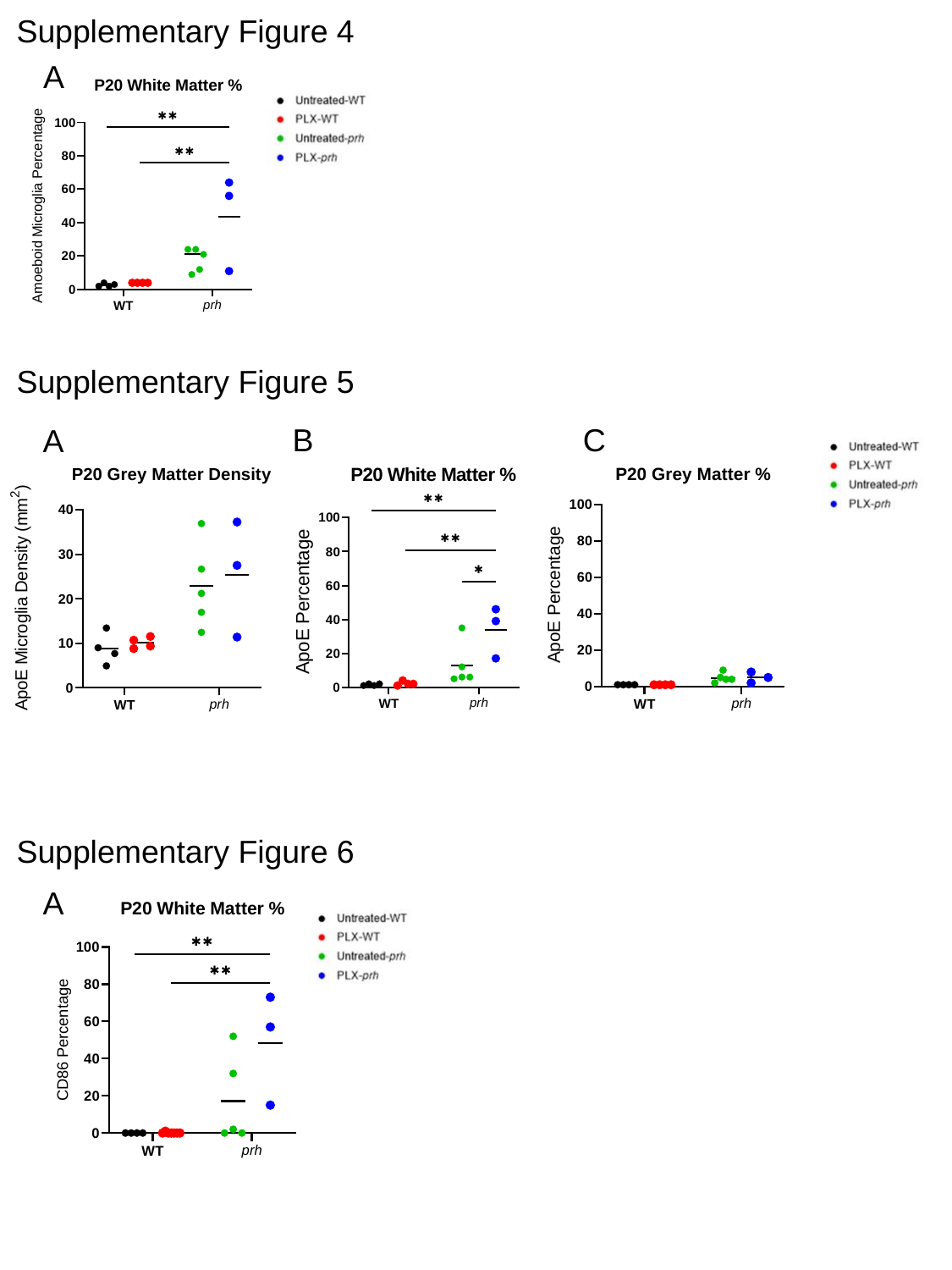

Supplementary Figure 4
A
Supplementary Figure 5
B
C
A
Supplementary Figure 6
A

## Slide 3
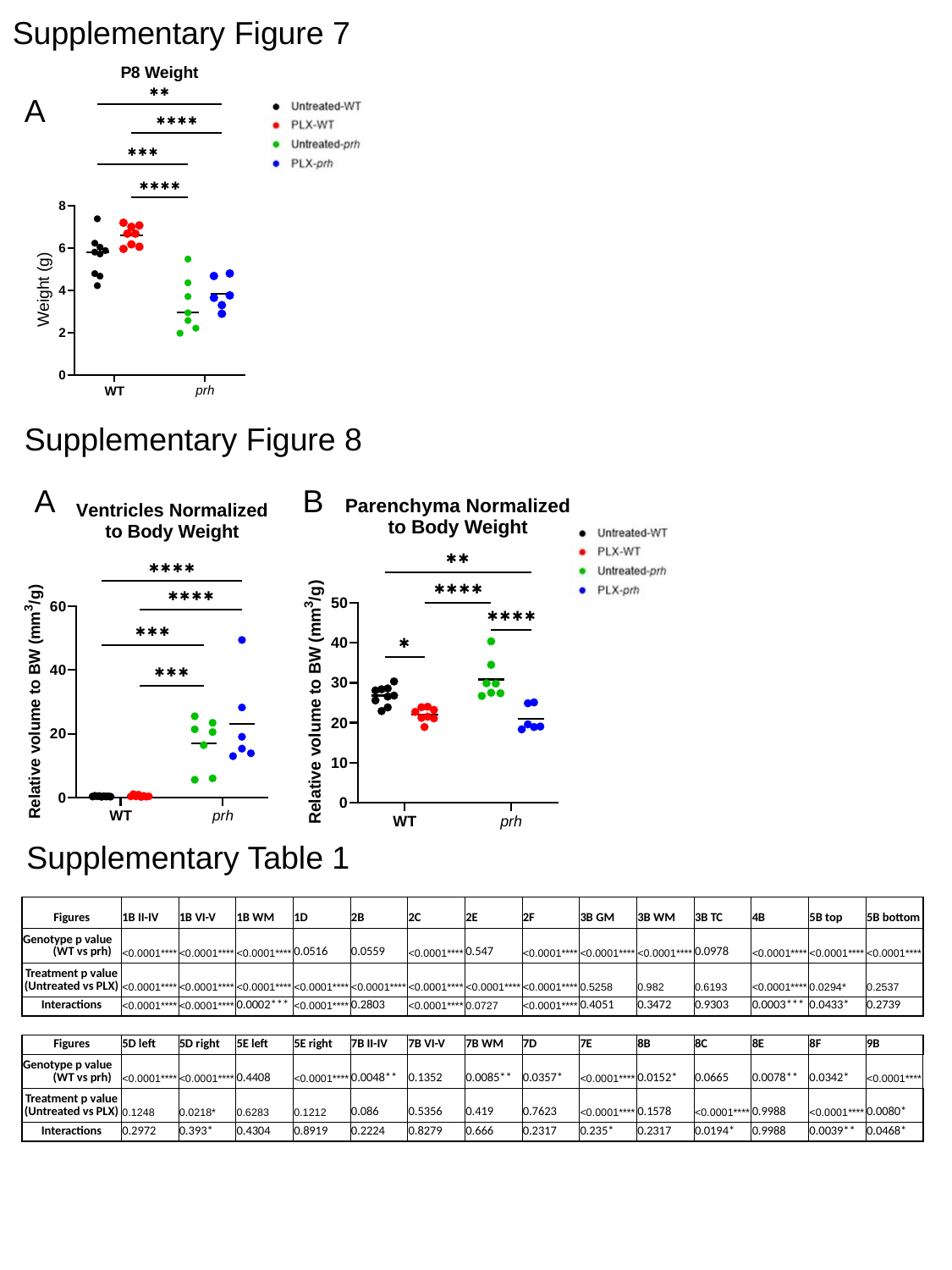

Supplementary Figure 7
A
Supplementary Figure 8
A
B
Supplementary Table 1
| Figures | 1B II-IV | 1B VI-V | 1B WM | 1D | 2B | 2C | 2E | 2F | 3B GM | 3B WM | 3B TC | 4B | 5B top | 5B bottom |
| --- | --- | --- | --- | --- | --- | --- | --- | --- | --- | --- | --- | --- | --- | --- |
| Genotype p value (WT vs prh) | <0.0001\*\*\*\* | <0.0001\*\*\*\* | <0.0001\*\*\*\* | 0.0516 | 0.0559 | <0.0001\*\*\*\* | 0.547 | <0.0001\*\*\*\* | <0.0001\*\*\*\* | <0.0001\*\*\*\* | 0.0978 | <0.0001\*\*\*\* | <0.0001\*\*\*\* | <0.0001\*\*\*\* |
| Treatment p value (Untreated vs PLX) | <0.0001\*\*\*\* | <0.0001\*\*\*\* | <0.0001\*\*\*\* | <0.0001\*\*\*\* | <0.0001\*\*\*\* | <0.0001\*\*\*\* | <0.0001\*\*\*\* | <0.0001\*\*\*\* | 0.5258 | 0.982 | 0.6193 | <0.0001\*\*\*\* | 0.0294\* | 0.2537 |
| Interactions | <0.0001\*\*\*\* | <0.0001\*\*\*\* | 0.0002\*\*\* | <0.0001\*\*\*\* | 0.2803 | <0.0001\*\*\*\* | 0.0727 | <0.0001\*\*\*\* | 0.4051 | 0.3472 | 0.9303 | 0.0003\*\*\* | 0.0433\* | 0.2739 |
| | | | | | | | | | | | | | | |
| Figures | 5D left | 5D right | 5E left | 5E right | 7B II-IV | 7B VI-V | 7B WM | 7D | 7E | 8B | 8C | 8E | 8F | 9B |
| Genotype p value (WT vs prh) | <0.0001\*\*\*\* | <0.0001\*\*\*\* | 0.4408 | <0.0001\*\*\*\* | 0.0048\*\* | 0.1352 | 0.0085\*\* | 0.0357\* | <0.0001\*\*\*\* | 0.0152\* | 0.0665 | 0.0078\*\* | 0.0342\* | <0.0001\*\*\*\* |
| Treatment p value (Untreated vs PLX) | 0.1248 | 0.0218\* | 0.6283 | 0.1212 | 0.086 | 0.5356 | 0.419 | 0.7623 | <0.0001\*\*\*\* | 0.1578 | <0.0001\*\*\*\* | 0.9988 | <0.0001\*\*\*\* | 0.0080\* |
| Interactions | 0.2972 | 0.393\* | 0.4304 | 0.8919 | 0.2224 | 0.8279 | 0.666 | 0.2317 | 0.235\* | 0.2317 | 0.0194\* | 0.9988 | 0.0039\*\* | 0.0468\* |
